# Testing of worn face mask and saliva for SARS-CoV-2

**DOI:** 10.3389/fpubh.2023.1237512

**Published:** 2023-09-18

**Authors:** Xiaoling Wang, Ohnmar Thwin, Zahin Haq, Zijun Dong, Lela Tisdale, Lemuel Rivera Fuentes, Nadja Grobe, Peter Kotanko

**Affiliations:** ^1^Renal Research Institute, New York, NY, United States; ^2^Icahn School of Medicine at Mount Sinai, New York, NY, United States

**Keywords:** COVID-19, SARS-CoV-2, hemodialysis, immunocompromised, viral shedding

## Abstract

**Background:**

Exhaled SARS-CoV-2 can be detected on face masks. We compared tests for SARS-CoV-2 RNA on worn face masks and matched saliva samples.

**Methods:**

We conducted this prospective, observational, case-control study between December 2021 and March 2022. *Cases* comprised 30 in-center hemodialysis patients with recent COVID-19 diagnosis. *Controls* comprised 13 hemodialysis patients and 25 clinic staff without COVID-19 during the study period and the past 2 months. Disposable 3-layer masks were collected after being worn for 4 hours together with concurrent saliva samples. ThermoFisher COVID-19 Combo Kit (A47814) was used for RT-PCR testing.

**Results:**

Mask and saliva testing specificities were 99% and 100%, respectively. Test sensitivity was 62% for masks, and 81% for saliva (*p* = 0.16). Median viral RNA shedding duration was 11 days and longer in immunocompromised versus non-immunocompromised patients (22 vs. 11 days, *p* = 0.06, log-rank test).

**Conclusion:**

While SARS-CoV-2 testing on worn masks appears to be less sensitive compared to saliva, it may be a preferred screening method for individuals who are mandated to wear masks yet averse to more invasive sampling. However, optimized RNA extraction methods and automated procedures are warranted to increase test sensitivity and scalability. We corroborated longer viral RNA shedding in immunocompromised patients.

## Introduction

Early diagnosis of coronavirus disease 2019 (COVID-19) is critical to limit the spread of severe acute respiratory syndrome coronavirus 2 (SARS-CoV-2). COVID-19 is primarily diagnosed though detection of viral RNA by reverse transcription-polymerase chain reaction (RT-PCR) in nasopharyngeal or oropharyngeal swabs. In a previous proof-of-principle study, we demonstrated that SARS-CoV-2 can be detected via RT-PCR on face masks (1). The feasibility of mask testing for SARS-CoV-2 has been corroborated by others (2–6).

Sensitivity and specificity of saliva testing for SARS-CoV-2 is comparable to nasopharyngeal or oropharyngeal swabs and has less variability (7, 8). Here we determined the diagnostic characteristics of SARS-CoV-2 RT-PCR in matched face masks and saliva samples and estimated the duration of SARS-CoV-2 RNA shedding.

## Methods

**Figure 1:**
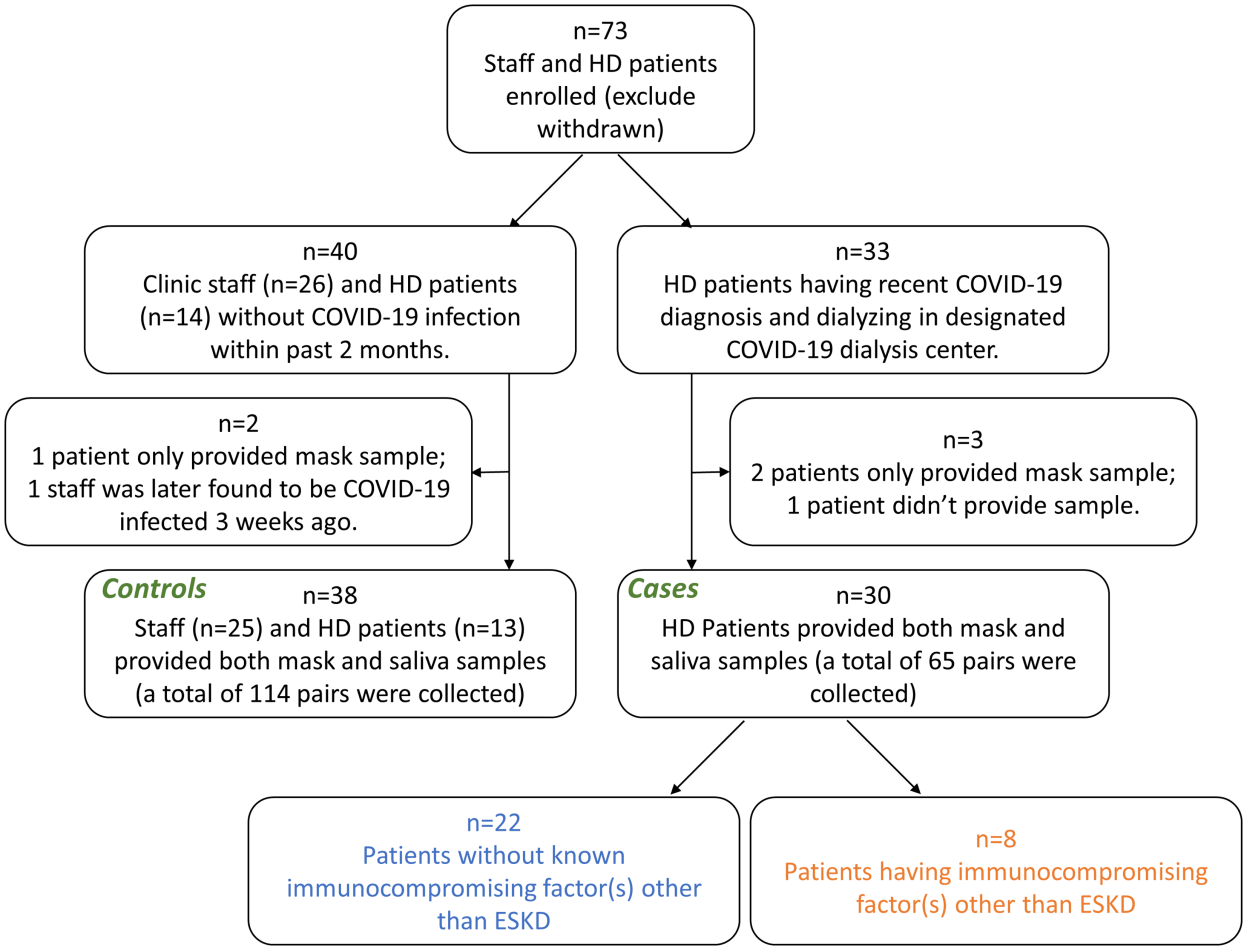
Flowchart of cohort selection. HD, hemodialysis; ESKD, end stage kidney disease.

Between December 2021, and March 2022, we conducted a prospective, observational, case-control study in two dialysis centers in New York City (approved by Western Institutional Review Board, protocol number 20213670) (Figure 1). All experiments were carried out in accordance with relevant guidelines and regulations. Informed consent was obtained from all subjects. *Controls* comprised 13 hemodialysis patients and 25 clinic staff without COVID-19 during the study period and the past 2 months. *Cases* comprised 30 hemodialysis patients with COVID-19, confirmed by positive nasopharyngeal RT-PCR testing.

**Figure 2:**
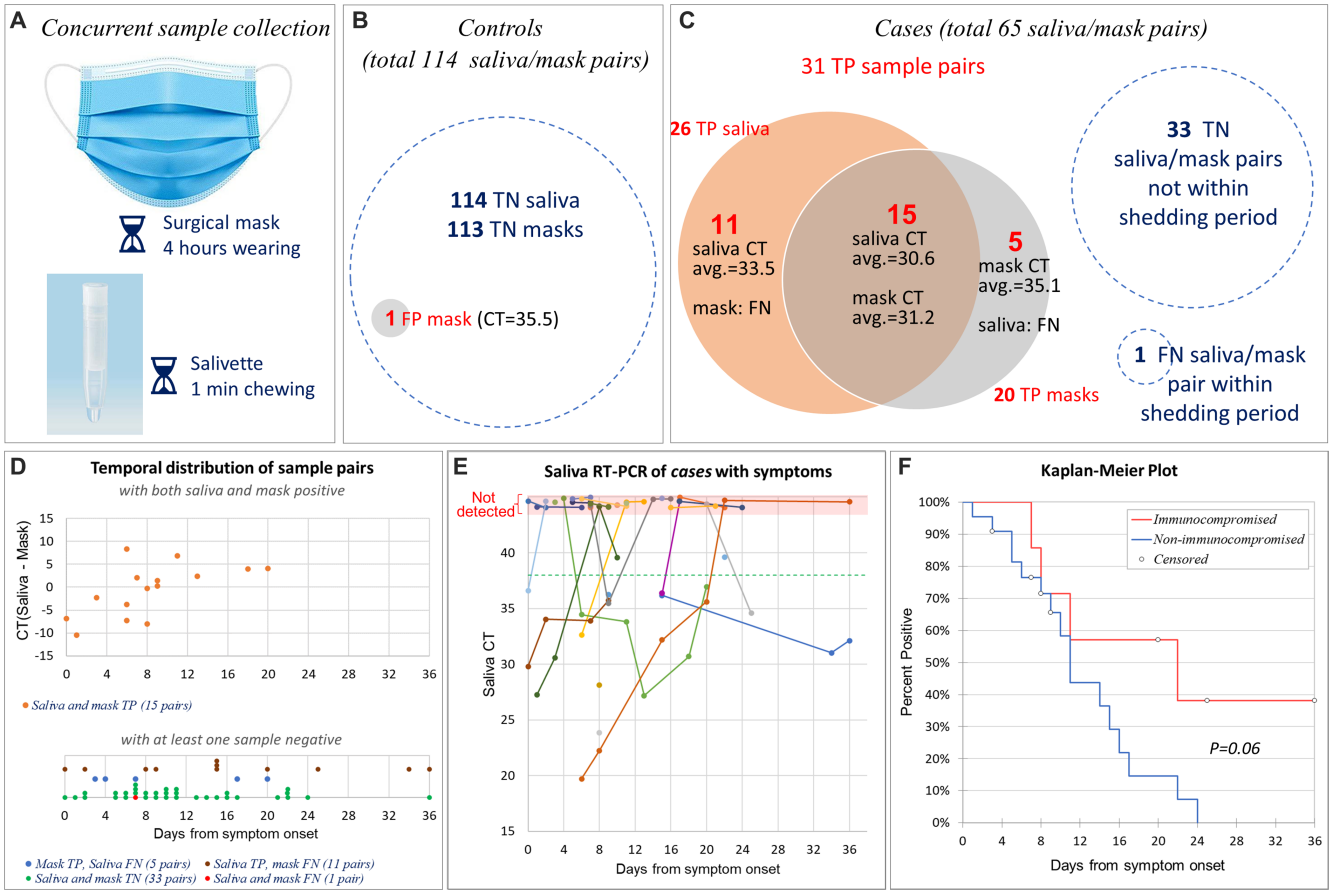
Sensitivity and specificity of SARS-CoV-2 mask and saliva testing. **(A)** Disposable 3-layer masks were collected after being worn for 4 h. Concurrently, saliva samples were collected via Salivette^®^ kit. **(B)** The *controls* provided 114 matched saliva/mask pairs. All 114 saliva samples and 113 mask samples tested true negative (TN) for SARS-CoV-2; one mask tested false positive (FP). **(C)** The *cases* provided 65 matched saliva/mask pairs. Among them, 31 pairs were true positive (TP), 33 pairs were true negative (TN), 1 pair was false negative (FN). True positive saliva and masks overlapped by 15 pairs. **(D)** Temporal distribution of 65 matched saliva/mask pairs after symptom onset (or initial PCR-diagnosis date for one asymptomatic patient). The upper panel comprised 15 pairs that tested TP in both saliva and masks. The *Y*-axis indicates the CT difference between saliva and mask. The lower panel comprises 50 pairs that tested negative in either the saliva test, mask test, or both. Note that *Y*-axis is not present in the lower panel. We have segregated the data of four groups and implemented point offsetting of overlapped points to ensure the visibility of all data points. **(E)** Saliva RT-PCR CT values of 29 symptomatic *cases* by days after symptom onset. Longitudinal samples from same patient were connected by straight line. The horizontal green dashed line indicates the CT cut-off for SARS-CoV-2 PCR. Non-detected CTs were indicated at the top pink area of the graph. Note that we slightly offset each dot in the pink area to enhance the visibility of those sharing the same dates. **(F)** Kaplan–Meier plot of 29 symptomatic cases, separated by patients with (*n* = 7) vs. without (*n* = 22) non-ESKD immunocompromising factors.

Disposable 3-layer masks were provided to subjects upon entering the dialysis center and collected into individual Ziplock^®^ bags after being worn for 4 h. Saliva samples were collected via Salivette^®^ (Sarstedt) at the time of mask collection (Figure 2A). There were no food or beverage restrictions imposed on the subjects; nevertheless, it is generally advised that subjects abstain from eating or drinking in the 15 min prior to sample collection. While acknowledging that actions such as talking, coughing, or sneezing by subjects could potentially impact sample positivity, we chose not to document these occurrences in order to maintain the protocol’s simplicity.

To be in alignment with the scenario of mask testing usability, samples were deposited into a collection box and accumulated over a day. Subsequently, they were transported to the laboratory once daily. The lab analysis was conducted the following morning, leading to a cumulative room temperature storage time of 13 to 24 h. The stability of samples stored at room temperature versus those stored at 4 degrees Celsius was not compared, due to the distinctive attributes of each mask and impossibility of duplicating a single mask. Mask RNA extraction was performed as described previously (1). Briefly, the inner mask surface was swabbed for 30 s using a cotton-tipped applicator that was pre-soaked with 100 μL PBS. The swab head was then placed in a microcentrifuge tube containing 600 μL PBS and vortexed for 10 min at 1000 rpm. Subjects’ mask or saliva RNA was extracted from 400 μL of above extracted solution or saliva using MagMax Viral/Pathogen kit (ThermoFisher A42352), respectively. Sars-CoV-2 RT-PCR reactions were performed using TaqPath RT-PCR COVID-19 Combo Kit (Thermo Fisher A47814). Bacteriophage MS2 was added to samples prior to RNA extraction. The multiplex probes anneal to one MS2 and three SARS-CoV-2 target sequences (S gene, N gene, ORF1ab). S gene target failure was observed in all study samples, indicating Omicron BA.1 infection (9). Samples with cycle threshold (CT) <38 for either N gene or ORF1ab were defined as SARS-CoV-2 positive. Samples lacking N gene and ORF1ab (CT not detectable or ≥38) but having MS2 amplification (CT <30) were defined as SARS-CoV-2 negative. The sample CT was calculated as the average of CT_N gene_ and CT_ORF1ab_.

The “shedding period” was defined as the days between COVID-19 symptom onset and the first date of two consecutive negative tests for SARS-CoV-2 in both saliva and masks. A positive test was true positive (TP) when obtained during the shedding period, otherwise false positive (FP). A negative test was true negative (TN) when observed outside the shedding period, otherwise false negative (FN). Test sensitivity [=TP/(TP + FN)] and specificity [=TN/(TN + FP)] are expressed as percentage. In *Cases*, sampling ceased after two consecutive negative tests.

The duration of SARS-CoV-2 RNA shedding period was estimated using Kaplan–Meier analysis.

## Results

Subjects’ demographic characteristics and vaccination status are presented in Table 1.

**Table 1:** Demographic characteristics and vaccination status of study subjects.

		Controls (13 HD patients and 25 staff)	Cases (30 HD patients)		
			Total	Non-immunocompromised	Immunocompromised
Subjects, *n*		38	30	22	8
Age (years)		46 ± 16 (23–86)	61 ± 14 (26–86)	61 ± 14 (32–82)	59 ± 14 (26–86)
Female, *n* (%)		23 (61%)	11 (37%)	8 (36%)	3 (38%)
Race	African American, *n* (%)	17 (45%)	22 (73%)	16 (73%)	6 (75%)
	White, *n* (%)	5 (13%)	7 (23%)	6 (27%)	1 (12%)
	Asian, *n* (%)	15 (39%)	1 (3%)	0	1 (12%)
	Native Hawaiian, *n* (%)	1 (3%)	0	0	0
COVID vaccination, *n* (%)		37 (98%)	25 (83%)	17 (77%)	8 (100%)

Age is reported as mean ± standard deviation and range. HD, hemodialysis.

### Sensitivity and specificity of SARS-CoV-2 testing

#### Specificity

The 38 *controls* provided 114 matched saliva/mask pairs (average 3 per subject; range 1 to 6). All saliva samples and 113 mask samples tested negative for SARS-CoV-2; one mask tested weakly positive for ORF1ab (CT = 35.5), resulting in specificities of 100% (saliva) and 99% (masks), respectively (Figure 2B).

#### Sensitivity

The 30 *cases* provided 65 matched saliva/mask pairs (average 2.2 per subject; range 1 to 7) 11 ± 8 days (range 0 to 36) after COVID-19 diagnosis, including 32 pairs collected during the shedding period. In one matched pair, both saliva and mask tested FN. Out of the remaining 31 pairs, 20 TP in masks, 26 TP in saliva, and 15 tested TP in both saliva and masks (Figure 2C), resulting in sensitivities of saliva and mask testing of 81% (95% CI, 64% to 93%) and 62% (95% CI, 44% to 79%), respectively. These sensitivities did not differ statistically (*p* = 0.16, two proportion *z* test with continuity correction).

Twenty-nine patients had mild to moderate symptoms with a well-defined date of onset. In them, COVID-19 was subsequently diagnosed by RT-PCR of nasopharyngeal swabs. One asymptomatic patient tested positive during the mandatory RT-PCR screening for new patients. Figure 2D illustrates the temporal distribution of all 65 matched saliva/mask pairs from 30 *cases* after symptom onset (or the PCR-diagnosis date for one asymptomatic patient). Among those 15 pairs that tested TP in both saliva and masks, overall CTs were comparable (mean CT_mask_ = 31.2; mean CT_saliva_ = 30.6; *p* = 0.66, paired two-sided *t*-test). Interestingly, there is a trend that saliva test was more sensitive at the beginning while mask test demonstrated higher sensitivity after 8 days from symptom onset (Figure 2D). As expected, in most patients, saliva viral RNA decreased over time (Figure 2E).

### Duration of the SARS-CoV-2 RNA shedding period

The asymptomatic patient was excluded from shedding period analysis because the start of his shedding period was undefined. Among the rest 29 symptomatic *cases*, none of the *cases* received Paxlovid^®^; all *cases* survived COVID-19. The median shedding period was 11 days (25th percentile: 8; 75th percentile 22) and independent of age, gender, vintage, and SARS-CoV-2 vaccination status (Cox proportional hazard model, results not shown).

Eight patients had immunocompromising factors other than end stage kidney disease (ESKD), comprising the excluded asymptomatic patient (#8) (Table 2). There was a trend towards longer SARS-CoV-2 RNA shedding in patients with (*n* = 7) vs. without (*n* = 22) non-ESKD immunocompromising factors (22 vs. 11 days; *p* = 0.06; Kaplan–Meier analysis, log-rank test; Figure 2F).

**Table 2:** Characteristics of patients with immunocompromising factor(s) other than ESKD.

Patient^#^	Sex	Race	COVID-19 vaccine brand	Number of Vaccine doses	Time since most recent COVID-19 vaccine (days)	Duration of shedding period (days)	Hospitalization due to COVID-19	Immunocompromising factor(s) other than ESKD and co-morbidities
1	M	Black	Moderna	3	91	>36 (censored)	Yes	Agammaglobulinemia
2	F	Black	Janssen & Moderna	2	71	>20 (censored)	No	SLE, hydroxychloroquine, MMF
3	F	Black	Pfizer	3	151	22	Yes	Heart failure, cardiomyopathy, NSTEM, liver transplant, HIV/ AIDS, hepatitis C, emtricitabine, dolutegravir, tenofovir
4	M	Black	Moderna	2	224	>25 (censored)	No	HIV/AIDS, Juluca (dolutegravir-rilpivirine)
5	M	Black	Moderna	3	71	8	No	Tacrolimus, valganciclovir
6	M	Asian	Moderna & Pfizer	3	38	11	No	Multiple myeloma
7	F	Black	Moderna	2	336	7	No	SLE, hydroxychloroquine
8	M	White	Pfizer	3	184	Not defined (asymptomatic)	No	Selective IgA deficiency

ESKD, end stage kidney diseases; M, male; F, female; SLE, systemic lupus erythematosus; MMF, mycophenolate mofetil; NSTEM, non-ST segment elevation myocardial infarction; HIV/AIDS, human immunodeficiency virus/acquired immunodeficiency syndrome.

## Discussion

While not statistically different, our study suggests directionally that testing for SARS-CoV-2 RNA in worn face masks is less sensitive compared to saliva testing (62% vs. 81%; *p* = 0.16). Still, mask testing may be a preferred screening method for individuals who are mandated to wear masks and are averse to invasive sampling, such as swabs.

Mask testing sensitivity can be improved by optimizing the RNA extraction process. Disposable mask fabric comprises non-woven polypropylene with electrostatic properties to enhance virus retention. We used 100 μL-PBS-soaked cotton-tipped applicators to vigorously swab the inner side for 30 s. This process likely failed to collect all viral RNA. Other publications suggested different ways to facilitate RNA extraction, such as attaching water soluble stripes to the mask inside (2, 5), or using Trizol (3) or DNA/RNA Shield (Zymo) (6). These methods and the use of more efficient and automated procedures may potentially increase the mask test sensitivity and efficiency. Of note, in five matched pairs all saliva samples tested FN while all masks tested TP, indicating that mask sampling may under certain circumstances be favorable. It’s generally considered that in the first week of COVID-19, RT-PCR positivity in breath sample and nasopharyngeal swab is comparable, and both have a sensitivity advantage over nasopharyngeal rapid antigen test (10).

In this study, a total of 12 masks and 6 saliva samples were identified as FN samples. The reason of false negativity encompasses several potential causes, including but not limited to diminished viral load, assay sensitivity, and variations in sample collection and viral material extraction. Notably, instances were observed where patients inadequately chewed the Salivette swab prior to its returning to the collection tube. Additionally, some patients may have worn masks loosely. Moreover, during the RNA extraction from masks, the region containing viral material might not have been comprehensively swabbed by the cotton-tipped applicator. Conversely, false positive results were infrequent, with a sole instance of a false positive mask provided by a clinical staff. This isolated case might be due to the cross-contamination during sample collection and handling, since this person was working in the dialysis center dedicated for COVID-positive HD patients.

In our *cases*, Omicron BA.1 was the most likely SARS-CoV-2 variant given an S gene target failure and concurrent New York City surveillance sequencing data (9, 11). In the general population, using RT-PCR in nasopharyngeal or oropharyngeal swabs, the median shedding period of Omicron variant was 11–17 days (12–14), comparable to the 11 days observed in our cohort of hemodialysis patients. We also corroborate the extended SARS-CoV-2 RNA shedding in immunocompromised patients (15).

A study limitation is the small *case* cohort (*N* = 30) that results in wide sensitivity confidence intervals. Whether or not the fact that our *cases* were hemodialysis patients limits generalizability is unclear; we are unaware of data suggesting a different virus exhalation rate in this population.

In conclusion, testing of worn face masks for exhaled SARS-CoV-2 RNA is feasible and highly specific. While its sensitivity is likely inferior to saliva testing, it should be explored further as a screening method, recognizing its operational ease, comfort, seamless repeatability, and lower cost.

## Data availability statement

The original contributions presented in the study are included in the article/supplementary material, further inquiries can be directed to the corresponding author.

## Ethics statement

The studies involving humans were approved by Western Institutional Review Board, protocol number 20213670. The studies were conducted in accordance with the local legislation and institutional requirements. The participants provided their written informed consent to participate in this study.

## Author contributions

XW, OT, ZH, ZD, LT, LF, NG, and PK contributed to the design and implementation of the clinical study. XW and ZH performed RNA extraction and RT-PCR. XW, OT, and PK analyzed the data. XW and PK wrote the manuscript with input from all authors. PK directed the project. All authors contributed to the article and approved the submitted version.

## Funding

This study was supported in part by National Institutes of Health/National Institute of Diabetes and Digestive and Kidney Diseases grant R01DK130067 and an award by the KidneyX COVID-19 Kidney Care Challenge, a joint effort of the U.S. Department of Health and Human Services (HHS) and the American Society of Nephrology.

## Conflict of interest

XW, OT, ZH, ZD, LT, LF, NG, and PK are employees of the Renal Research Institute, a wholly owned subsidiary of Fresenius Medical Care. PK holds stock in Fresenius Medical Care.

## Publisher’s note

All claims expressed in this article are solely those of the authors and do not necessarily represent those of their affiliated organizations, or those of the publisher, the editors and the reviewers. Any product that may be evaluated in this article, or claim that may be made by its manufacturer, is not guaranteed or endorsed by the publisher.
